# Cytotoxic Screening and Enhanced Anticancer Activity of *Lippia alba and Clinopodium nepeta* Essential Oils-Loaded Biocompatible Lipid Nanoparticles against Lung and Colon Cancer Cells

**DOI:** 10.3390/pharmaceutics15082045

**Published:** 2023-07-29

**Authors:** Boris Rodenak-Kladniew, María Agustina Castro, Rocío Celeste Gambaro, Juan Girotti, José Sebastián Cisneros, Sonia Viña, Gisel Padula, Rosana Crespo, Guillermo Raúl Castro, Stephan Gehring, Cecilia Yamil Chain, Germán Abel Islan

**Affiliations:** 1 INIBIOLP—Instituto de Investigaciones Bioquímicas de La Plata (UNLP-CONICET LA PLATA), Facultad de Ciencias Médicas UNLP, La Plata 1900, Argentina; magustinacg@gmail.com (M.A.C.); juangirotti@yahoo.com.ar (J.G.); 2 IGEVET—Instituto de Genética Veterinaria (UNLP-CONICET LA PLATA), Facultad de Ciencias Veterinarias UNLP, La Plata 1900, Argentina; rociogambaro@gmail.com (R.C.G.); gpadula@igevet.gob.ar (G.P.); 3 INIFTA—Instituto de Investigaciones Fisicoquímicas Teóricas y Aplicadas (UNLP-CONICET LA PLATA), La Plata 1900, Argentina; sebacisneros@gmail.com (J.S.C.); yamil.chain@gmail.com (C.Y.C.); 4 CIDCA—Centro de Investigación y Desarrollo en Criotecnología de Alimentos (UNLP-CONICET LA PLATA), Facultad de Ciencias Exactas UNLP, La Plata 1900, Argentina; sonia_via@hotmail.com; 5 IFEC—Instituto de Farmacología Experimental de Córdoba (UNC-CONICET UNC), Facultad de Ciencias Químicas UNC, Córdoba 5000, Argentina; ro_crespo@yahoo.com; 6 Nanomedicine Research Unit (Nanomed), Center for Natural and Human Sciences (CCNH), Universidade Federal do ABC (UFABC), Santo André 09210-580, Brazil; grcastro@gmail.com; 7 Children’s Hospital, University Medical Center of the Johannes, Gutenberg University, Langenbeckstr. 1, 55131 Mainz, Germany; stephan.gehring@uni-mainz.de; 8 CINDEFI—Centro de Investigación y Desarrollo en Fermentaciones Industriales, Laboratorio de Nanobiomateriales (UNLP-CONICET LA PLATA), Facultad de Ciencias Exactas UNLP, La Plata 1900, Argentina

**Keywords:** essential oils, solid lipid nanoparticles, cancer cells, drug delivery, biocompatibility, anticancer mechanisms

## Abstract

Plant and herbal essential oils (EOs) offer a wide range of pharmacological actions that include anticancer effects. Here, we evaluated the cytotoxic activity of EO from *Lippia alba* (chemotype linalool), *L. alba* (chemotype dihydrocarvone, LaDEO), *Clinopodium nepeta* (L.) *Kuntze* (CnEO), *Eucalyptus globulus*, *Origanum* × *paniculatum*, *Mentha* × *piperita*, *Mentha arvensis* L., and *Rosmarinus officinalis* L. against human lung (A549) and colon (HCT-116) cancer cells. The cells were treated with increasing EO concentrations (0–500 µL/L) for 24 h, and cytotoxic activity was assessed. LaDEO and CnEO were the most potent EOs evaluated (IC_50_ range, 145–275 µL/L). The gas chromatography–mass spectrometry method was used to determine their composition. Considering EO limitations as therapeutic agents (poor water solubility, volatilization, and oxidation), we evaluated whether LaDEO and CnEO encapsulation into solid lipid nanoparticles (SLN/EO) enhanced their anticancer activity. Highly stable spherical SLN/LaDEO and SLN/CnEO SLN/EO were obtained, with a mean diameter of 140–150 nm, narrow size dispersion, and Z potential around −5mV. EO encapsulation strongly increased their anticancer activity, particularly in A549 cells exposed to SLN/CnEO (IC_50_ = 66 µL/L CnEO). The physicochemical characterization, biosafety, and anticancer mechanisms of SLN/CnEO were also evaluated in A549 cells. SLN/CnEO containing 97 ± 1% CnEO was highly stable for up to 6 months. An increased in vitro CnEO release from SLN at an acidic pH (endolysosomal compartment) was observed. SLN/CnEO proved to be safe against blood components and non-toxic for normal WI-38 cells at therapeutic concentrations. SLN/CnEO substantially enhanced A549 cell death and cell migration inhibition compared with free CnEO.

## 1. Introduction

Cancer is among the leading causes of death in most countries worldwide and is estimated to be responsible for 19.3 million new cases and 10 million deaths in 2020 [1]. The burden of cancer incidence and mortality is expanding rapidly, with a 47% increase in new cancer cases (28.4 million) projected for 2040 [1]. By far, lung cancer is the leading cause of cancer death (1.8 million), followed by liver (830,000), stomach (780,000), breast (685,000), and colon (575,000) cancer. The expansion of precision medicine and innovative therapeutic approaches, including targeted therapy and immunotherapy, in the last few years has provided excellent results in some instances. However, a wide range of constraints, such as ineffectiveness, resistance, unfavorable side effects, and extremely expensive costs, have lowered expectations for these treatments [2]. This scenario highlights the necessity to investigate new therapeutic options and/or improve the established ones.

Nature is an excellent source of molecules for innovation in drug discovery. More than 50% of all anticancer drugs approved in the world between 1940 and 2010 were natural products or their derivatives [3,4]. Among natural products in general and phytochemicals in particular, essential oils (EOs) have gained special interest because of their broad diversity of bioactivities [4].

Essential oils are complex, lipid-soluble, and volatile chemicals produced by aromatic plants that act as secondary metabolites. They are natural mixtures (20–60 compounds) characterized by high concentrations (20–70%) of two or three main components, particularly terpenes and terpenoids [5,6,7]. Essential oils are obtained from the leaves, roots, and stems of plants by hydrodistillation, steam distillation, or dry distillation, and from fruit peels by cold pressing [7]. In nature, they play a critical function in plant defense against bacteria, fungi, insects, and herbivores, as well as pollinating insect attractants [5,6,7].

Since ancient times, EOs have been used for the prevention and treatment of a variety of diseases. Their chemopreventive and chemotherapeutic effects have been widely documented. In addition, in vitro studies have demonstrated that EO extracts employed as single agents selectively target cancer cells, with no or significantly reduced cytotoxicity against healthy cells [6,7,8]. The complex mixtures found in EO compositions should be considered an advantage because the potential multi-target and synergistic effects of the individual components are in accordance with the goal of cancer treatments to evade resistance mechanisms [9,10,11]. Notwithstanding the anticancer benefits described, there are several drawbacks to using EO, including degradation by oxidation and evaporation, decomposition by ultraviolet light and heat, and low water solubility [12].

The nanoencapsulation of EO into lipid nanoparticles appears to be a strategy to solve these issues [13,14,15]. Solid lipid nanoparticles (SLN) emerged in the 1990s for the encapsulation of lipophilic drugs as an alternative to conventional lipid colloidal carriers such as emulsions and liposomes [15,16]. The primary benefits of SLN include their high physical stability, high drug loading, controlled drug release profile, surface modification to enhance passive and active cell targeting, and protection of labile drugs from environmental degradation [17,18,19]. SLNs are biocompatible, non-toxic, and easily produced on a large scale [19]. Moreover, the inclusion of liquid lipids (such as EO) into the solid lipid matrix of SLN promotes their reorganization into nanostructured lipid carriers (NLC) [20,21], improving their stability, encapsulation efficiency, and drug loading capacity, and limiting the expulsion of cargo molecules during storage [17,18,19].

The present study aimed to identify the most active EO obtained from eight different plants and herbs against lung and colon cancer cells; it aimed to characterize the most active EO and develop from them EO-loaded SLNs as a biocompatible delivery system to improve their anticancer activities; and it aimed to explore the stability, encapsulation efficiency, kinetic release profile, biocompatibility, and cytotoxic and antimetastatic activities of the most promising formulation.

## 2. Materials and Methods

### 2.1. Materials

The solid lipid myristyl myristate (MM) was generously provided by Croda (Buenos Aires, Argentina). Poloxamer 188 (Pluronic^®^ F68), tetrazolium dye MTT [3-(4,5-dimethylthiazol-2-yl)-2,5-diphenyltetrazolium bromide], Dulbecco’s modified eagle medium (DMEM), and penicillin–streptomycin (P/S) were obtained from Gibco (Invitrogen Corporation, Grand Island, NY, USA). Fetal bovine serum (FBS) was obtained from Internegocios (Buenos Aires, Argentina). Other reagents were of analytical grade from Carlo Erba (Milan, Italy), Merck (Darmstadt, Germany), or equivalent brands and used as received.

### 2.2. Plant Material and EO Extraction

The leaves and stems of cultivated *Lippia alba* (chemotype linalool), *L. alba* (chemotype dihydrocarvone), *Clinopodium nepeta* (L.), *Kuntze* subsp. spruneri (Boiss.), *globulus*, *Origanum* × *paniculatum*, *Mentha* × *piperita*, *Mentha arvensis* L., and *Rosmarinus officinalis* L. were harvested from the Experimental Station of the School of Forestry and Agricultural Sciences, UNLP (La Plata, Buenos Aires, Argentina). The samples were identified by Prof. Nestor Bayón (Cátedra de Sistemática Vegetal, School of Forestry and Agricultural Sciences, UNLP). EOs were extracted and quantified by hydrodistillation and conserved as previously reported [22]. Voucher specimens No. F16 for *L. alba* (chemotype linalool), No. F17 for *L. alba* (chemotype dihydrocarvone), No. F19 for *C. nepeta*, No. F33 for *E. globulus*, No. F20 for *Origanum* × *paniculatum*, No. F24 for *Mentha* × *piperita*, No. F1 for *M. arvensis* L., and No. F31 for *R. officinalis* L. are deposited in the Bank of Essential Oils, Laboratory of Phytochemistry, School of Forestry and Agricultural Sciences, UNLP.

### 2.3. Cell Culture

Human colorectal carcinoma HCT-116 (CCL-247) and lung adenocarcinoma A549 (CCL-185) cells were provided by the American Type Culture Collection (ATCC). WI-38 cells (normal human embryonic lung fibroblasts, ATCC CCL-75) were a kind gift from Dr. Natalia Scaglia (School of Medicine, National University of La Plata). Cells were cultured in DMEM (Gibco) supplemented with 10% FBS and 1% P/S (Gibco) at 37 °C under a 5% CO_2_ atmosphere.

### 2.4. Cytotoxic Screening of Different EOs

The cytotoxic activity of EO was determined by the MTT assay [23]. HCT-116 (5 × 10^3^) and A549 (5 × 10^3^) cells were placed in a 96-well microplate and kept for 24 h at 37 °C and 5% CO_2_. Afterward, the medium was discarded and replaced with increasing concentrations (0–500 µL/L) of the eight mentioned EO dissolved in 100% ethanol (final concentration < 0.1%) in 10% FBS-supplemented DMEM for 24 h. After rinsing with PBS, cells were incubated with MTT solution (0.5 mg/mL in serum-free DMEM) for 3 h. The produced formazan was dissolved in 100 µL DMSO, the plates were shaken for 10 min, and the absorbance at λ = 560 nm was measured in a microplate reader (Beckman Coulter DTX 880, USA). Cell viability was expressed as a percentage of the untreated control (100% survival).

### 2.5. Analysis by Gas Chromatography–Mass Spectrometry

The volatile organic compound (VOC) composition of EO was analyzed with a Hewlett-Packard 6890 gas chromatograph (GC) coupled to a mass-selective detector (Agilent 5975C VL) as previously described [22]. Briefly, VOC separation was performed employing a ZB-5HT Inferno column (30 m, 0.25 mm d.i., 0.25 μm film, Phenomenex, Torrance, CA, USA). The injector was operated at 250 °C. The oven was programmed as follows: 40 °C for 1 min; 10 °C/min to 300 °C with a holding time of 3 min at the final temperature. The MSD was set at 70 eV and operated in scan mode with a mass range of 35–600 amu; transference line at 300 °C; ionization chamber at 250 °C; and quadrupole at 150 °C. The samples were obtained by head space-solid phase microextraction (HS-SPME). A preconditioned 65 μm polydimethylsiloxane/divinylbenzene fiber (PDMS/DVB, Supelco, Bellefonte, PA, USA) was exposed for 2 min to 1 μL of each EO placed in a 2 mL glass vial sealed with a Teflon cover with a rubber seal at 25 °C. VOC were identified by interpretation of their mass fragmentation pattern; spectra were also compared to data from MS libraries (NIST 05 Mass Spectral Library) and the literature (Adams, 2007). VOC chain lengths were confirmed by calculating their Kovats index (KI).

### 2.6. Preparation of EO-Loaded SLN (SLN/EO)

The SLN/EO were prepared through homogenization by the ultrasonication method, as previously reported by our group [24]. In a pre-warmed water bath at 70 °C, 200 mg MM (2.0% *w*/*v*) was first melted, and then 250 µL EO was added. Ten milliliters of a pre-warmed aqueous solution of poloxamer 188 (3.0% wt) were added to the lipidic phase. Rapidly, the pre-emulsion was subjected to ultrasonication for 10 min (40% amplitude) in a Cole-Parmer ultrasonic processor (130 W, USA). For control experiments, empty SLNs were prepared following the same procedure but omitting the addition of EO.

### 2.7. Transmission Electron Microscopy (TEM)

The TEM analysis was carried out in a Jeol-1200 EX II-TEM microscope (Jeol, MA, USA) as described earlier [20].

### 2.8. Particle Size, Zeta Potential, and Polydispersity Index

Nanoparticle mean diameter, size distribution (polydispersity index, PDI), and zeta potential (Z-pot) were measured in a Nano ZS Zetasizer Instrument (Malvern Instruments Corp., UK) at 25 °C, as previously described [25].

### 2.9. Cytotoxic Activity of Selected Free and Encapsulated EO

HCT-116 (5 × 10^3^) and A549 (5 × 10^3^) cells were plated in 96-well plates for 24 h under standard conditions and treated with increasing concentrations (25–400 µL/L EO) of free and/or encapsulated EO for 24 h. Cell viability was determined by MTT, as described earlier. This range (25–400 µL/L EO) was chosen based on previous results to cover non-toxic (25 µL/L) to highly cytotoxic (400 µL/L EO) concentrations of LaDEO and CnEO in both HCT-116 and A549 cells, therefore evidencing the potential enhancement of anticancer activity after encapsulation.

### 2.10. Clinopodium Nepeta (L.) Kuntze (CnEO) Detection

The detection of CnEO was carried out through ultraviolet-visible (UV–Vis) spectroscopy at λ_max_ = 258 nm. The CnEO UV–Vis spectra and calibration curves were conducted in the range of 0.01 to 0.1 µL/L CnEO dissolved in 20% ethanol in PBS 10 mM (pH 7.4) or Ac-AcH 10 mM (pH 5.0).

### 2.11. Encapsulation Efficiency (EE)

Percent EE for SLN/CnEO was determined indirectly by calculating the amount of non-encapsulated CnEO [20] by UV–Vis spectroscopy (λ_max_ = 258 nm). Percent EE was calculated as:

(1) $$EE\left(\%\right)=\frac{Q0-\left(Cr\times V\right)}{Q0}\times 100$$

where Q_0_ = initial quantity of CnEO; Cr = concentration of CnEO in the filtered solution; and V = total volume.

The same procedure was followed for the empty SLN to discard possible interferences in UV–Vis detection.

### 2.12. Physical Stability

The SLNs were placed at 4 °C, protected from light, for six months, and their stability was followed by analyzing changes in mean particle size, PDI, and Z-pot.

### 2.13. Release Studies

CnEO release was determined as previously described [21] using two different buffer systems: phosphate buffer (pH 7.4) and acetate/acetic acid buffer (AC- + AcH, pH 5.0). The CnEO concentration was measured at λ_max_ = 258 nm using a UV–Vis spectrophotometer (Shimadzu, Japan).

### 2.14. Hemotoxicity Studies

Heparinized venous blood from healthy donors was used after obtaining the corresponding written informed consent. The blood was placed in a six-well plate in Ham F12 culture medium containing 10% FBS and exposed to increasing amounts of SLN, free-CnEO, and SLN/CnEO (100–400 µL/L CnEO) at 37 °C for 24 or 48 h. After centrifuging the mixture at 2500× *g* for 5 min, the precipitate was discarded. The proportion of lysed red blood cells was quantified by measuring the released hemoglobin at λ = 540 nm. Hemolysis (100%) was determined by exposing erythrocytes to 1.0% Triton X-100, while the negative control was obtained by incubating erythrocytes in phosphate-buffered saline (PBS).

### 2.15. Cytotoxic Activity of SLN/CnEO on Normal Lung WI-38 Fibroblasts

WI-38 cells were plated in 96-well plates at a density of 8 × 10^3^ cells/well for 24 h under standard conditions and treated with concentrations of SLN/CnEO (50 and 100 µL/L CnEO) that significantly inhibited A549 lung cancer cells. Cell viability was determined by MTT, as described earlier.

### 2.16. Evaluation of Mitochondrial Membrane Potential (MMP)

A549 cells (5 × 10^3^) seeded in a 96-well plate were exposed to CnEO or SLN/CnEO (50, 100, and 200 µL/L) or 0.5 mM hydrogen peroxide (positive control) for 3 or 24 h. Then, MMP was measured using the fluorescent dye rhodamine-123, as previously reported [26].

### 2.17. Cell Death

A549 cells (2 × 10^4^) were placed in a 24-well plate and allowed to adhere for 24 h. Then, cells were exposed to 0.1% ethanol (Control), CnEO (50 and 100 µL/L), or SLN/CnEO (50 and 100 µL/L CnEO) dissolved in DMEM for 24 h. Cell death was measured by the trypan blue exclusion assay, as reported earlier [26].

### 2.18. Inhibition of Cell Migration

A549 cells (7.5 × 10^4^) were seeded in 24-well plates for 24 h. The monolayers were scratched with a sterile pipette tip (200 µL) on the midline of the well and washed with DMEM to remove the detached cells. Then, the cells were exposed to DMEM containing 0.1% ethanol (Control), CnEO (50 and 100 µL/L), or SLN/CnEO (50 and 100 µL/L CnEO). A549 cell migration was assessed employing a Fluorescence Microscope (Olympus LX71 Inverted Tokyo, Japan) at 0 and 48 h. The wound healing area was defined employing Image J software (1.53k, NIH, USA) [26].

### 2.19. Statistical Analysis

Experimental data are expressed as means ± standard deviation (SD). Data were analyzed using one-way analysis of variance (ANOVA), the Tukey–Kramer multiple-comparison test (significance level set at *p* < 0.05), or the unpaired *t*-test. Cell viability Non-linear regression curves (SigmaPlot software 14.0; Systat Software, Inc., Point Richmond, CA, USA) were used to calculate the IC_50_ values for cell viability.

## 3. Results and Discussion

### 3.1. Cytotoxic Activity of EO against Lung A549 and Colon HCT-116 Cells

The cytotoxic activity of the eight EOs was assessed by exposing A549 and HCT-116 cancer cells to increasing EO concentrations (0–500 µL/L) and calculating their IC_50_ values (Table S1). *L. alba* (chemotype dihydrocarvone) essential oil (LaDEO) and CnEO were the two most active EO in both cell lines, inhibiting cell growth in a concentration-dependent manner (IC_50_ LaDEO = 275 and 145 µL/L; IC_50_CnEO = 205 and 200 µL/L in A549 and HCT-116 cells, respectively). Except for *L. alba* chemotypes linalool (IC_50_ = 400 µL/L) and *globulus* (IC_50_ = 500 µL/L) in HCT-116 cells, IC_50_ values were >500 µL/L in all cases. Based on these results, LaDEO and CnEO were selected for further studies.

### 3.2. Chemical Composition of EO

LaDEO and CnEO chemical compositions (compounds higher than 1.0%) were analyzed (Table 1). The complete CnEO composition was previously reported by our group [22]. As expected, most of the compounds identified were monoterpenes. The major constituents of LaDEO were dihydrocarvone isomer 1 (29.6%), limonene (25.2%), and dihydrocarvone isomer 2 (23.8%). It has been reported that within the *L. alba* species, limonene is frequently present as one of the major compounds of EO, accompanied by at least one of the five monoterpenic ketones, including dihydrocarvone [27].

On the other hand, pulegone (37.2%), menthone (26.6%), and isomenthone (11.7%) were the three most abundant monoterpenes found in CnEO. Božovic et al. [28] reported that at least three types of CnEO can be recognized. The most abundant components consist of C-3 oxygenated p-menthanes such as pulegone, menthone, isomenthone, and piperitone. Among them, pulegone is the main variant and the major component associated preferentially with menthone and/or isomenthone [28].

Our findings on EO composition are in line with those expected for LaDEO and CnEO according to literature data.

### 3.3. Synthesis of SLN/EO

The lipid matrix for nanoparticle synthesis used was MM, considering the lipophilic nature of the main VOCs, LaDEO and CnEO. MM is appropriate for the development of stable colloidal nanoparticles, as shown by earlier studies from our group [24].

**Figure 1:**
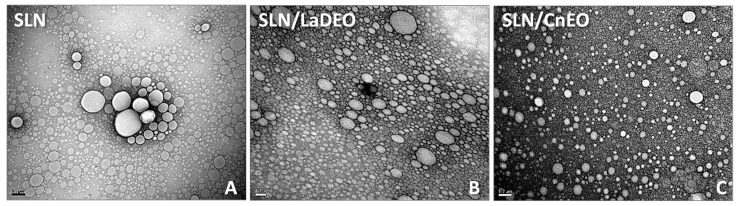
TEM images of SLN (**A**), SLN/LaDEO (**B**), and SLN/CnEO (**C**). Scale bar: 200 nm.

A stable emulsion of EO in SLN was produced. TEM pictures revealed the existence of uniform and spherical nanoparticles (Figure 1). Compared to empty SLN, the incorporation of CnEO into the MM matrix appeared to result in smaller nanoparticles.

**Figure 2:**
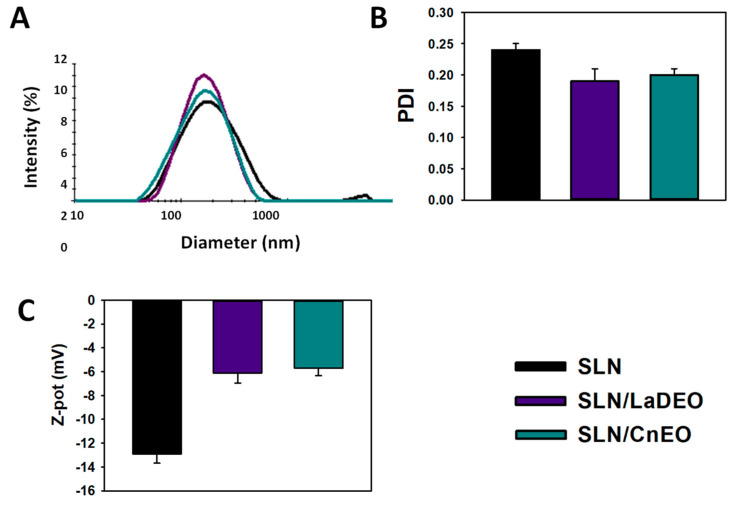
DLS analysis. (**A**) Particle size distribution, (**B**) Polydispersity index (PDI), and (**C**) Z-potential (Z-pot). The results express the mean ± SD (n = 3).

The outcomes of the TEM images were confirmed by a dynamic light scattering (DLS) analysis (Figure 2). For nanoparticles dispersed in aqueous environments, DLS is a more suitable and less-invasive technique than TEM since it reflects the presence of agglomerates and aggregates in a more representative way (indicated by the PDI parameter). The mean diameters of SLN, SLN/LaDEO, and SLN/CnEO were 147, 151, and 141 nm, respectively. The PDI was ≤0.3 in all cases, indicating high compatibility for biomedical purposes [29]. Indeed, SLN/LaDEO and SLN/CnEO displayed a PDI ≤ 0.2, whereas SLN presented a PDI of 0.24. These findings may be attributed to the good emulsion dispersion but also suggest that EO inclusion would promote a reorganization in the SLN structure [20,21,24]. This observation became even clearer when a population of SLN particles larger than 1000 nm vanished after being combined with EO (Figure 1 and Figure 2A), highlighting the monodispersed nature of the SLN/EO formulations. A decrease in the Z-pot of SLN from −12 mV to −5/6 mV was found after the incorporation of EO, which further reinforces the idea that EO would be crucial for SLN restructuring.

### 3.4. Cytotoxic Activity of Free and Encapsulated LaDEO and CnEO

**Figure 3:**
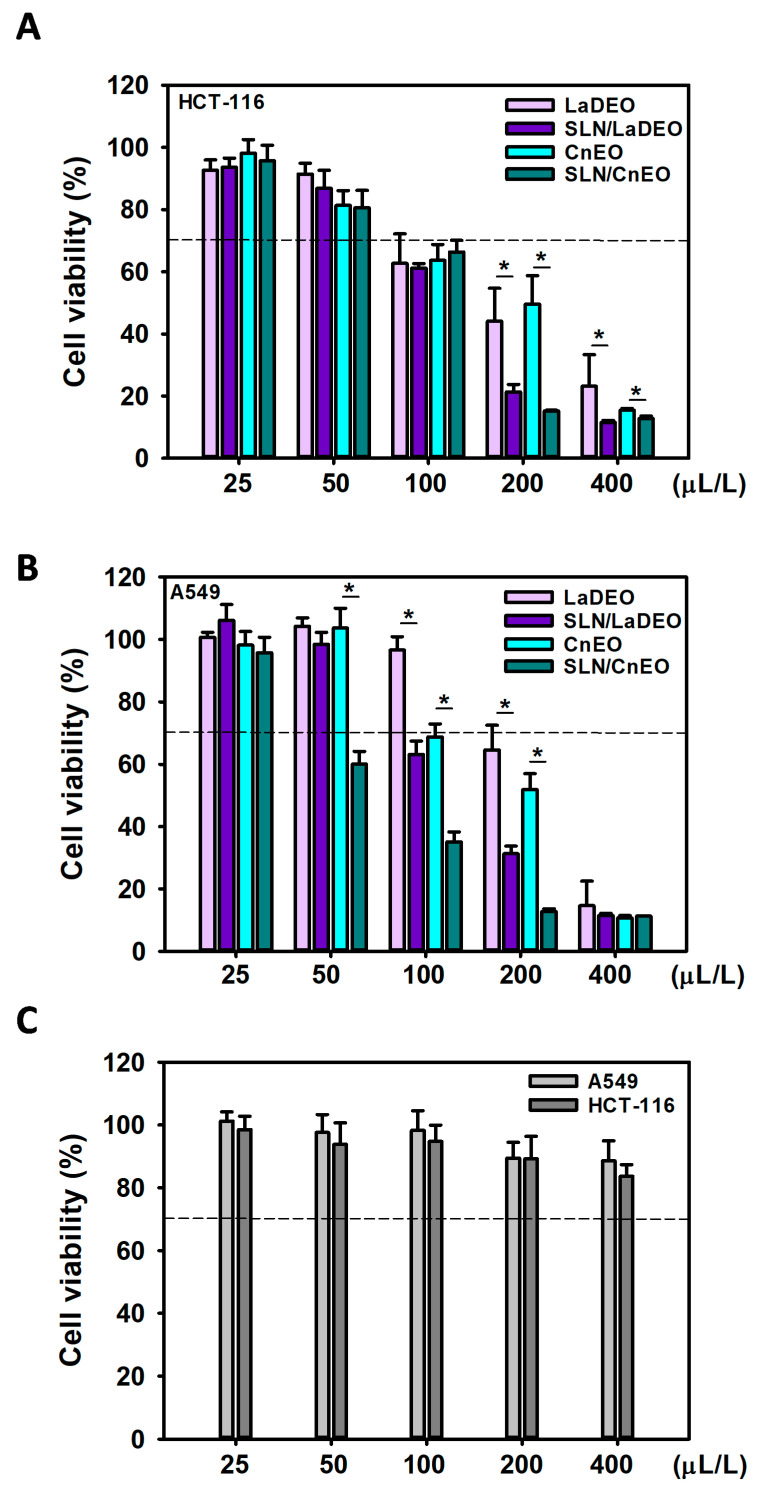
Cytotoxicity of SLN/EO in colon cancer (HCT-116) and lung cancer (A549) cells. HCT-116 (**A**) and A549 (**B**) cells were exposed to increasing concentrations of free and encapsulated EOs or equivalent quantities of empty SLNs (**C**), and cell viability was evaluated by the MTT assay after 24 h. Free EO was dissolved in ethanol (0.1% *v*/*v* max). Results are expressed as means ± SD (n = 6) compared to control cells (ethanol 0.1%, 100% viability), (*) *p* < 0.001 (70% viability is indicated by the dotted line; below that, the treatment could be considered cytotoxic).

As shown in Section 3.1, LaDEO and CnEO were the most active EOs against lung A549 and colon HCT-116 cells. Nevertheless, EO employment in pharmaceutical products has some restrictions due to its high volatility, low aqueous solubility, chemical instability, and low bioavailability [30]. Intending to determine the potential advantages of EO encapsulation into SLN, the cytotoxic activity of free and SLN/EO (SLN/LaDEO and SLN/CnEO) was explored in A549 and HCT-116 cells. The cells were incubated with increasing amounts of free and encapsulated EO (25–400 µL/L) and equivalent amounts of unloaded SLN for 24 h (Figure 3). 

Free EO and SLN/EO decreased cell viability in a concentration-dependent manner in both cell lines. Encapsulation of LaDEO and CnEO increased their cytotoxicity in HCT-116 cells from 200 µL/L, where cell viability decreased from 44.0 and 49.6% (free EO) to 21.3 and 15.4% (SLN/EO), respectively (Figure 3A; *p* < 0.001 in both cases). The effect of EO nanoencapsulation against A549 cells was most important since a significantly enhanced cytotoxic activity was observed from 50 µL/L CnEO and 100 µL/L LaDEO (Figure 3B). A549 cell viability decreased from 96.5 to 63.2% and from 64.6 to 31.3% after encapsulation of 100 µL/L and 200 µL/L LaDEO, respectively (*p* < 0.001). CnEO incorporation into SLN decreased cell viability from 103.4 to 60.6% at 50 µL/L, and from 68.8 to 35.1% at 100 µL/L, respectively (*p* < 0.001). Empty SLN in equivalent quantities did not produce any cytotoxicity at all in any of the two cell lines (Figure 3C) [31].

The improvement of EO activity by encapsulation may be attributed to a series of aspects such as extended EO stability in the culture medium at 37 °C, augmented EO permeation through the cell membrane, the sustained presence of cytotoxic amounts of EO resulting from a controlled release from SLN, possible evasion of expulsive mechanisms, and rapid metabolism of the main bioactive VOC due to SLN protection [32,33,34].

The IC_50_ values of SLN/LaDEO and SLN/CnEO in A549 and HCT-116 cells are shown in Table 2.

Based on these findings, the most promising system (SLN/CnEO against A549 cells) was explored for further studies.

### 3.5. CnEO Encapsulation and Release and SLN/CnEO Stability

**Figure 4:**
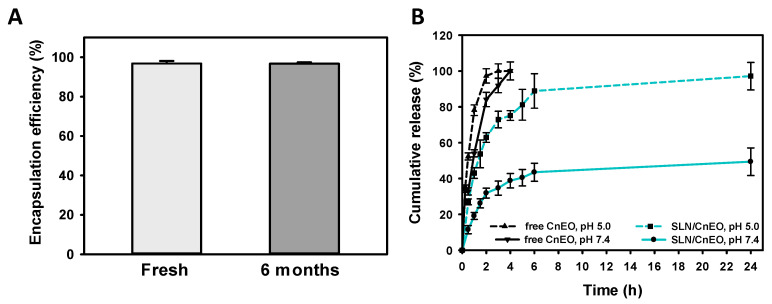
CnEO EE and cumulative release. (**A**) CnEO EE (%). (**B**) CnEO release from SLN/CnEO at pH 7.4 and 5.0. Values are expressed as the mean ± SD (n = 3).

The EE of CnEO into SLN was assessed, as previously reported by our group [20]. A UV–Vis scanning of CnEO was performed (230–270 nm), observing a stable and intense peak at 258 nm (Figure S1). Interestingly, CnEO’s UV–Vis spectrum and λ_max_ = 258 nm quite coincided with those of its main component, pulegone [35].. CnEO EE in SLN reached a high value of approximately 97%, which was maintained for at least 6-month storage (Figure 4A). The high amounts of CnEO encapsulated were shown to correlate with those of VOCs such as 1,8-cineole or linalool, previously encapsulated by our group [20,24].

The kinetic release of CnEO from the SLN matrix was also determined (Figure 4B). To mimic the physiological conditions and the acidic environment found in the endolysosomal compartments, a pH-dependent release of CnEO was tested at pH = 7.4 and 5.0 [21,36]. The free diffusion of CnEO through the dialysis membrane was also evaluated under both conditions. First, it was observed that the SLN/CnEO formulation displayed a conventional bi-phasic release profile at both pHs. An initial robust burst release during the first 4 h was followed by a sustained and slow release for up to 24 h. Then, the CnEO kinetic release from SLN showed a clear pH dependence. At neutral pH, only 43% of the total CnEO was released after 6 h, whereas about 90% was released at acidic pH during the same time. After 24 h, CnEO was almost completely released from SLN at pH = 5.0, whereas only 49% was at pH = 7.4. Additionally, free CnEO was completely released after 4 h at both pHs. Altogether, these findings may explain the reduced hemotoxicity of encapsulated CnEO in comparison with free CnEO at physiological pH. Furthermore, CnEO intracellular release would be favored into acidic endosomes/lysosomes following cellular uptake through the endocytic pathway, described as the primary mechanism of SLN internalization [37].

To assure the formulation’s reproducibility and potential uses, SLN/CnEO stability is a crucial factor. Stability was examined after 6 months of 4 °C storage in terms of EE (Figure 4A), size, PDI, and Z-pot. No significant changes in the examined parameters were found, as would be expected for this type of system [38,39,40], indicating that SLN/CnEO was stable over this period.

### 3.6. SLN/CnEO Biocompatibility

One of the primary objectives of cancer therapy is to kill tumor cells by preventing, or at least minimizing, detrimental side effects on healthy organs.

**Figure 5:**
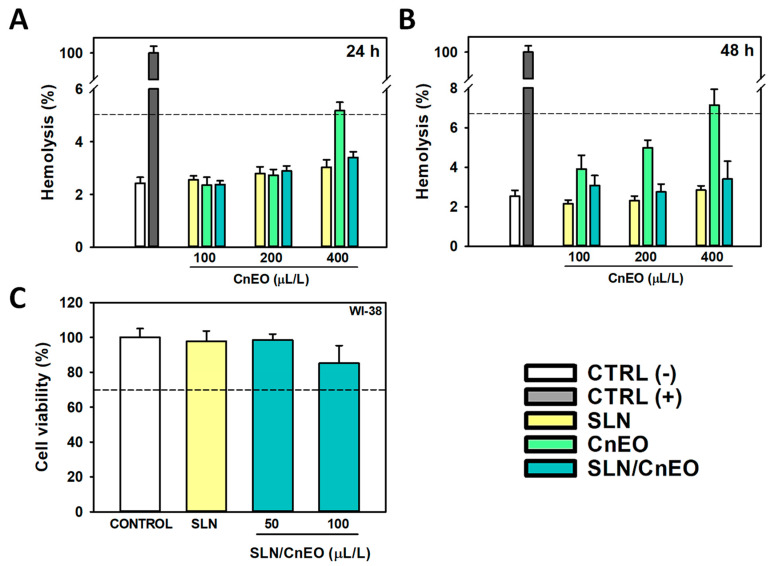
Biocompatibility of SLN/CnEO. (**A**,**B**) Hemotoxicity of empty SLN, free CnEO, and SLN/CnEO. Hemotoxicity of 100, 200, and 400 µL/L CnEO and equivalent doses of empty SLN and SLN/CnEO were evaluated after 24 h (**A**) and 48 h (**B**) of exposure. Results are expressed as the mean ± SD (n = 3). (**C**) (−): untreated; (**C**) (+): 1% Triton X-100-lysed erythrocytes. (**C**) Effect of SLN/CnEO on normal lung WI-38 fibroblasts. Cells were exposed to SLN/CnEO (50 and 100 µL/L), and cell viability was evaluated by the MTT assay after 24 h. Results are expressed as the mean ± SD (n = 6). Of the viability, 70% is indicated by the dotted line; below that, the treatment could be considered cytotoxic.

The SLN/CnEO hemotoxicity was tested as a first step in identifying the potential negative effects of the formulation (Figure 5A,B). The interaction with erythrocytes becomes an important factor when assessing the safety of nanoparticles, particularly if intravenous delivery is thought of as an SLN potential route [41]. According to the ISO/TR 7406 standard, biomaterials that result in a hemolytic ratio of less than 5% can be deemed safe for biomedical uses [42]. Here, erythrocytes were exposed to very high concentrations (100–400 µL/L) of free or encapsulated CnEO and long exposure periods (up to 48 h) to identify the safety of the formulations. A dose-dependent hemotoxicity of free CnEO was observed, which was significant at the highest tested concentration (400 µL/L) (Figure 5A,B). On the other hand, hemolytic ratios of less than 3.5% were found at any condition analyzed in SLN/CnEO. Probably, SLN may be protecting erythrocytes from direct CnEO exposure, where the main VOCs could be destabilizing their membranes, leading to hemolysis.

In addition, the cytotoxicity of SLN/CnEO on WI-38 normal lung fibroblasts was examined. Figure 5C shows that SLN/CnEO was non-toxic for WI-38 cells (viability > 70%) at 50 and 100 µL/L CnEO, which inhibited about 40 and 65% A549 cell viability, respectively (Figure 3B). This specific activity dependent on the cell type (normal or tumoral) may be associated with the selective activity of monoterpenes against cancer cells previously described [26,43], although further studies are required to shed light on this issue.

Altogether, these findings suggest that SLN/CnEO is a biocompatible and safe system able to improve the anticancer activity of CnEO.

### 3.7. Anticancer Mechanisms of CnEO and SLN/CnEO

Essential oils and their major VOCs, monoterpenes, were shown to exert cytostatic, cytotoxic, and antimetastatic effects, leading to the inhibition of cancer cell progression [6,44]. One of the main mechanisms by which EO and conventional anticancer drugs induce cell death depends on their ability to depolarize the mitochondrial membrane, leading to apoptosis and/or autophagic cell death [6]. In addition, the permeabilization of the mitochondrial membrane caused by the convergence of numerous death signal transduction pathways results in the release of pro-apoptotic factors into the cytosol, such as cytochrome C, triggering apoptotic cell death [45]. We have previously shown that mitochondrial membrane depolarization is one of the earliest events involved in monoterpene-induced cell cycle arrest and/or cell death in A549 [26] and other cancer cell types [46,47]. Here, we evaluated A549 cell death and mitochondrial membrane depolarization after exposure to free and encapsulated CnEO (Figure S2). It was observed that free CnEO did not increase cell death at the tested concentrations (50 and 100 µL/L CnEO) after 24 h of incubation. In contrast, CnEO encapsulation strongly enhanced A549 cell death from 1.3 and 3.4% to 12.6 and 15.9% at 50 and 100 µL/L CnEO, respectively (*p* < 0.001).

On the other hand, the mitochondrial membrane potential of A549 cells exposed to CnEO and SLN/CnEO was not altered at concentrations up to 200 µL/L CnEO, neither at short (3 h) nor long (24 h) time incubations, suggesting that other mechanisms apart from MMP depolarization may be involved in A549 cell death, such as death-ligand-induced apoptosis, autophagy, or necroptosis [48,49,50,51].

Due to the highly heterogeneous composition of EO as well as the wide variety of cancer types, defining an exclusive mechanism of action becomes challenging. Initially, research focused on exploring the antioxidant and anti-inflammatory properties of EO, suggesting their potential for treating cancer. Cancer cells are characterized by a moderate increase in reactive oxygen species (ROS) compared to normal cells. Moreover, inflammation is also considered a hallmark of cancer [4,50,51]. On the other hand, EO, or their main VOC, may also exhibit anticancer effects by increasing ROS generation [26,47,51]. It is well established that ROS modulate signaling pathways involved in cell survival and proliferation, such as MAP kinases and Akt/mTOR, as well as EO and VOC, and are capable of directly interfering with these pathways, leading to cancer cell death [47,50,51]. In a previous study, we found that CnEO and *L. alba* (chemotype linalool) essential oils did not present significant antioxidant capacity but, in contrast, demonstrated pro-oxidant activity on low-density lipoproteins (LDL) [22]. Here, we found that CnEO encapsulation enhanced cell viability inhibition and promoted cell death independently of MMP collapse; however, further studies are required to shed light on the antiproliferative mechanisms exerted by SLN/CnEO.

**Figure 6:**
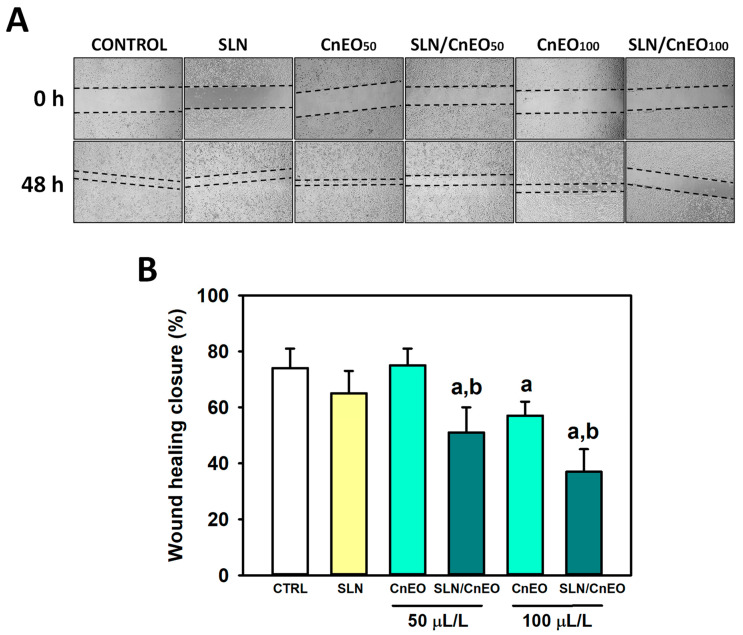
Encapsulation of CnEO increases the inhibition of A549 cell migration. Cell migration was analyzed by the wound healing assay. Cells were exposed to 0.1% ethanol (control), empty SLN, free CnEO, or SLN/CnEO (50 and 100 µL/L) for 48 h. (**A**) Representative images were obtained at 0 and 48 h (40×). (**B**) Quantitative analysis of wound healing closure. Data are presented as the mean ± SD (n = 4). (a) *p* < 0.05 vs. Control; (b) *p* < 0.05 vs. equivalent concentration of free CnEO.

Cell migration, a feature of tumor spread and cancer invasion, is a key aspect to consider. Indeed, lung cancer is among the malignancies that most commonly metastasize [47]. Bioactive compounds with cytotoxic and anti-metastatic properties are ideal candidates. Therefore, the impact on cell migration was investigated using the wound healing assay to better understand both the anti-cancer mechanisms of free CnEO and the advantages of its encapsulation in SLN (Figure 6). We observed that 100 µL/L CnEO inhibited cell migration to some degree (*p* < 0.001), whereas SLN/CnEO impaired the migration of A549 cells at lower concentrations (50 µL/L) and significantly improved the anti-migratory effects of free-CnEO (Figure 6B, *p* < 0.001 vs. control cells and free-CnEO), suggesting that CnEO had antimetastatic effects on lung A549 cells, which are definitely exacerbated after its encapsulation into SLN.

## 4. Conclusions

Our findings revealed that LaDEO and CnEO were the most active among eight different EOs against both lung cancer (A549) and colon cancer (HCT-116) cells. Encapsulation of LaDEO and CnEO into SLN improved the anticancer effects of free EO in both cell lines. SLN/CnEO in A549 cells resulted in the most effective system. A deeper characterization of SLN/CnEO revealed a high EE of CnEO and an interesting pH dependency of CnEO release from SLN, favoring CnEO release in acidic conditions, which mimicked the endolysosomal compartment. SLN/CnEO seemed to be highly biocompatible since no toxic effects on red blood cells or normal lung fibroblasts were observed at concentrations at which A549 cancer cells were completely killed. In contrast to free CnEO, SLN/CnEO induced significant A549 cell death that seemed to be independent of the mitochondrial depolarization pathway and substantially enhanced the inhibition of A549 cell migration mediated by free CnEO.

The obtained formulations could be considered green anticancer tools in adjuvant and/or complementary therapies. However, additional investigations focusing on molecular mechanisms and in vivo studies are recommended. This information may be useful for the development of innovative treatment modalities, including the incorporation of conventional lipophilic chemotherapeutics into SLN/EO.

## Figures and Tables

**Table 1:** LaDEO and CnEO chemical composition.

Volatile Organic Compound (VOC)	KI ^a^	Chemical Composition (%)	
		LaDEO ^b^	CnEO ^c^
Myrcene	993	3.38	-
3-Octanol	996	-	2.43
Limonene	1036	25.23	-
cis-Sabinene hydrate	1074	-	4.21
Linalool	1106	1.33	1.25
Menthone	1165	-	26.59
Isomenthone	1178	-	11.71
Isomenthol	1182	-	4.61
trans-Isopulegone	1188	-	3.16
Terpinen-4-ol	1188	-	2.41
Dihydrocarvone isomer 1	1217	29.64	-
Dihydrocarvone isomer 2	1225	23.81	-
Pulegone	1255	-	37.22
Carvol	1261	1.40	-
1-Cyclohexanone, 2-methyl-2-(3-methyl-2-oxobutyl)	1299	-	1.15
β-Elemene, (-)-	1405	1.27	-
β-Caryophyllene	1439	2.13	-

(a) KI: Kovats index. (b) LaDEO: *Lippia alba* (chemotype dihydrocarvone) essential oil. (c) CnEO: *Clinopodium nepeta* essential oil.

**Table 2:** IC50 values of SLN/LaDEO and SLN/CnEO on A549 and HCT-116 cells.

	IC50 (µL/L)	
Formulation	A549	HCT-116
SLN/LaDEO	131 ± 8	122 ± 10
SLN/CnEO	66 ± 5	134 ± 11

Dose–response curves were obtained by nonlinear regression, and the IC_50_ values were calculated. Data are expressed as the means ± SD. Each experiment was carried out in triplicate.

## Data Availability

The data that support the findings of this study are available from the corresponding authors, B.R-K. and G.A.I., upon request.

## Supplementary Materials

The following supporting information can be downloaded at: https://www.mdpi.com/article/10.3390/pharmaceutics15082045/s1, Figure S1: (A) UV-Vis scanning of CnEO (230–270 nm). A stable and intense peak at 258 nm was observed. (B–C) Calibration curve of CnEO (0.01–0.1 µL/L) in (B) 20% EtOH in PBS 10 mM (pH 7.4) or (C) 20% EtOH Ac-AcH 10 mM (pH 5.0); Figure S2: Encapsulation of CnEO increases A549 cell death. Table S1: IC50 values of eight different essential oils on A549 and HCT-116 cells. Table S1. IC50 values of eight different essential oils on A549 and HCT-116 cells.
