# Supplementary material for: Cytotoxic Screening and Enhanced Anticancer Activity of Lippia alba and Clinopodium nepeta Essential Oils-Loaded Biocompatible Lipid Nanoparticles against Lung and Colon Cancer Cells
Source: Pharmaceutics. 2023 Jul 29;15(8):2045. doi: 10.3390/pharmaceutics15082045 (PMC10459614; doi:10.3390/pharmaceutics15082045)
Supplement: Supplementary file 1 [file pharmaceutics-15-02045-s001.zip › pharmaceutics-2522902-supplementary.pdf]

FIGURE S1

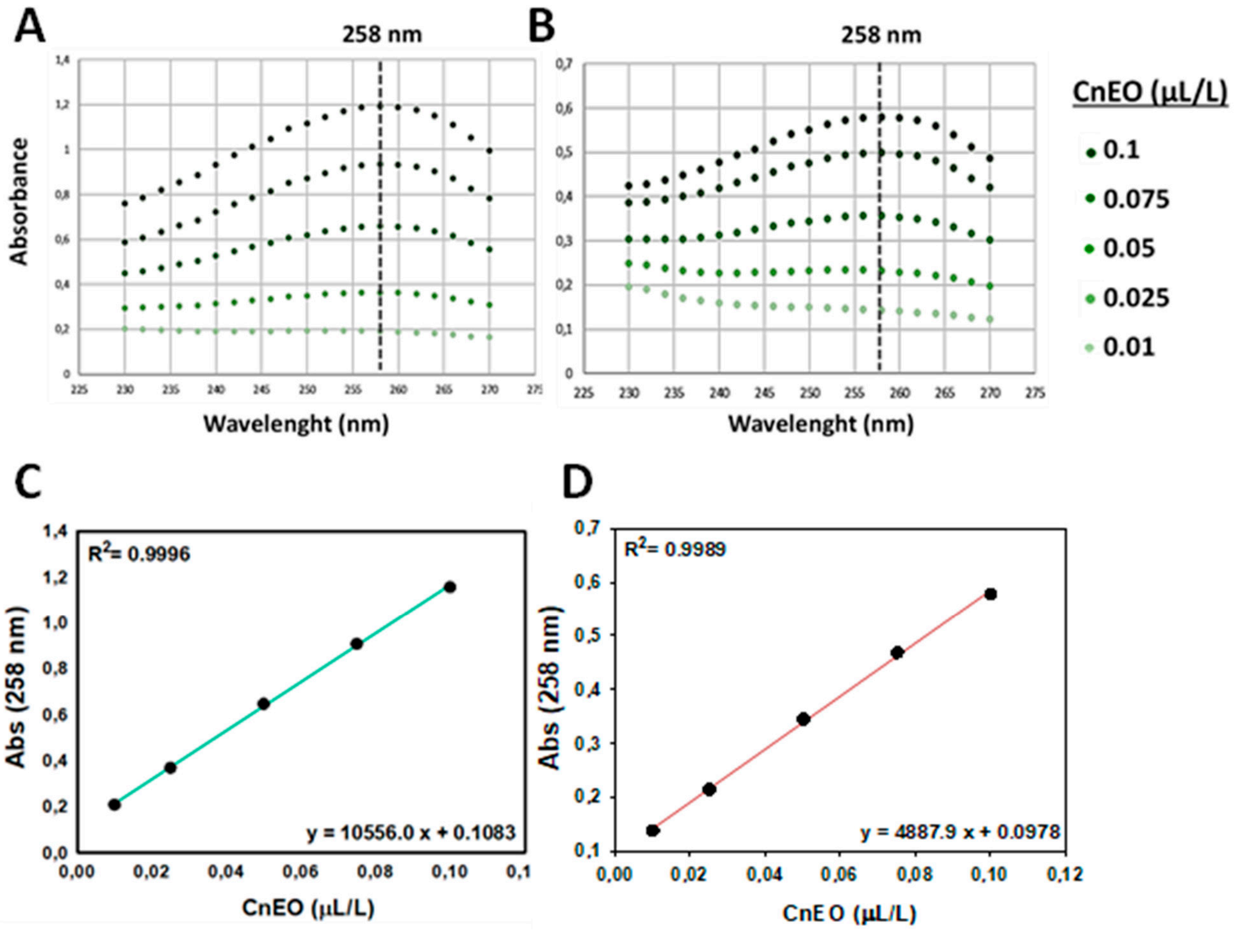

Figure S1. (A) UV-Vis scanning of CnEO (230-270 nm). A stable and intense peak at 258 nm was observed. (B-C) Calibration curve of CnEO (0.01-0.1 µL/L) in (B) 20% EtOH in PBS 10 mM (pH 7.4) or (C) 20% EtOH Ac-AcH 10 mM (pH 5.0).

FIGURE S2

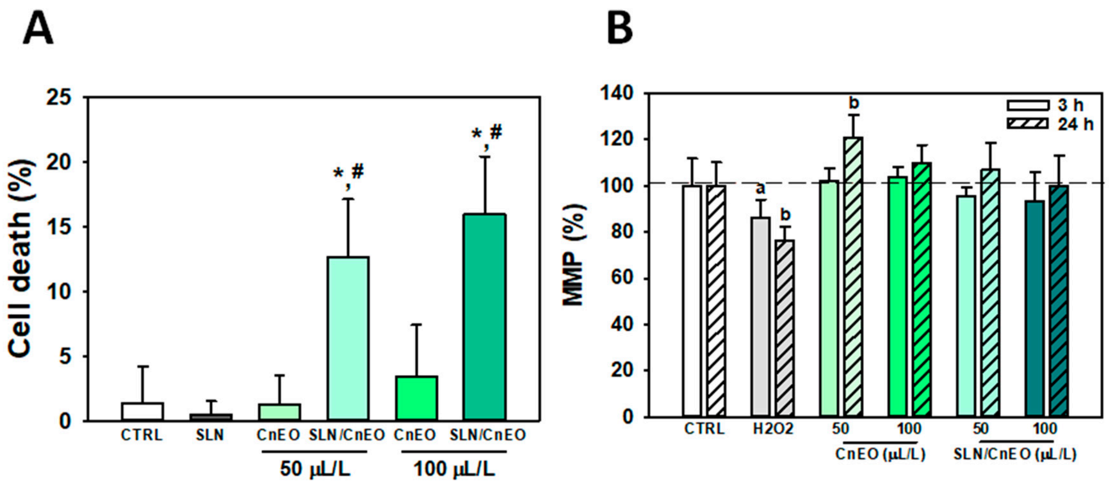

Figure S2. Encapsulation of CnEO increases A549 cell death and cell migration inhibition. (A) Cells were incubated with 0.1% ethanol (Control), empty SLN (0.8 mg MM/ml), CnEO or SLN/CnEO (50 and 100 µL/L CnEO) for 24 h, and cell death was assessed by trypan blue staining. (*) $p < 0.05$ vs. Control; (#) $p < 0.05$ vs. equivalent concentration of free CnEO. (B) Cells were incubated with 0.1% EtOH (Control), 0.5 mM H2O2 (positive control), or 1.0 mM SLN/CnEO (50, 100, and 200 µL/L CnEO) for 3 or 24 h and then stained with rhodamine-123. Data are expressed as percentage MMP loss compared with control cells. Results are normalized to living cells and data are presented as means ± SD (n=4). (a) $p < 0.05$ vs. Control (3 h); (b) $p < 0.05$ vs. Control (24 h).

TABLE S1

Table S1. IC50 values of eight different essential oils on A549 and HCT-116 cells

		IC50 (µL/L)	
**Number**	**Essential Oil**	**A549**	**HCT-116**
1	*L. alba (linalool)*	> 500	400 ± 24
2	*L. alba (dihydrocarvone)*	275 ± 26	145 ± 31
3	*Clinopodium nepeta (L) Kuntze*	205 ± 11	200 ± 28
4	*Eucaliptus globulus*	> 500	~500
5	*Mentha piperita*	> 500	> 500
6	*Origanum × paniculatum*	> 500	> 500
7	*Mentha arvensis*	> 500	> 500
8	*Rosmarinus officinalis*	> 500	> 500

Dose–response curves were obtained by nonlinear regression and the IC50 values calculated. Data are means ± SD. Each experiment was carried out in triplicate.
